# Psychological Burdens among Teachers in Germany during the SARS-CoV-2 Pandemic—Subgroup Analysis from a Nationwide Cross-Sectional Online Survey

**DOI:** 10.3390/ijerph19159773

**Published:** 2022-08-08

**Authors:** Clemens Koestner, Viktoria Eggert, Theresa Dicks, Kristin Kalo, Carolina Zähme, Pavel Dietz, Stephan Letzel, Till Beutel

**Affiliations:** 1 Institute of Occupational, Social and Environmental Medicine, University Medical Center of the Johannes Gutenberg University of Mainz, Obere Zahlbacher Str. 67, 55131 Mainz, Germany; 2 Department of Sports Medicine, Disease Prevention and Rehabilitation, Johannes Gutenberg University Mainz, Albert-Schweitzer-Strasse 22, 55128 Mainz, Germany; 3 Institute for Teachers’ Health, University Medical Center of the Johannes Gutenberg University of Mainz, Kupferbergterrasse 17–19, 55116 Mainz, Germany

**Keywords:** COVID-19, mental health, teachers, school, depression, anxiety, burnout

## Abstract

Background: Schools underwent massive changes during the SARS-CoV-2 pandemic worldwide. Besides existing occupational health challenges, teachers had to deal with biological and psychological burdens that had the potential to impact their psychological well-being. The aim of the present study was to (i) assess the current state of psychological burdens in German teachers and (ii) identify highly burdened subgroups to derive and address interventions. Methods: A nationwide cross-sectional online survey was conducted among teachers at all school types in Germany in March 2021. Data on psychological strains were assessed using established (e.g., PHQ-4) and new—pandemic-specific—(e.g., COVID-19-associated anxieties) instruments. ANOVAs and Tukey’s post hoc tests were used to identify highly burdened subgroups (e.g., gender, age, and number of risk factors for severe courses of COVID-19) of teachers. Results: Psychological burdens in German teachers (N = 31.089) exceeded the level of the general population, for example, regarding symptoms of depression (PHQ-2, M = 1.93 vs. 1.24) or generalized anxiety (GAD-2, M = 1.72 vs. 1.03). Subgroup analysis revealed that psychological burdens were unevenly distributed among different groups of teachers; for example, younger teachers (18–30 years) showed more depression symptoms compared with their older colleagues (56–67 years) (PHQ-2, M = 2.01 vs. 1.78). Conclusions: The online survey was conducted during the “third wave” of SARS-CoV-2 in Germany, which might have influenced risk perception and psychological strains. Future studies at different times, ideally longitudinal monitoring of the mental health of teachers, are recommended. Based on our results, evidence-based subgroup-specific interventions should be implemented to sustain teachers’ mental health; for example, younger teachers or teachers with risk factors for a severe course of COVID-19 should receive special attention and support. Teachers from special needs schools whose mental health is, on average, good could also be a starting point for identifying the health promotion structural elements of this school type (e.g., fewer students per teacher). However, beyond the specific pandemic-related psychological burdens, the classic occupational health challenges of physical, biological, and chemical stress and their resulting strains should not be disregarded.

## 1. Background

The ongoing SARS-CoV-2 pandemic has shown the potential to act as a catalyst for a variety of existing somatic, psychological, and social burdens worldwide. Furthermore, it has generated new ones. Several international studies analyzing the general population have provided evidence that, in various countries, alarming associations between the SARS-CoV-2 pandemic and psychological burdens (e.g., stress, anxiety, depression, insomnia, or anger) emerged. This is in line with findings about responses to other, previous outbreaks of infections, for example, the 2015 MERS-CoV outbreak in Korea [1]. In a Chinese study that included 17,865 participants, various negative emotions (e.g., anxiety, depression, or indignation) increased, whereas the sources of positive emotions (happiness) and life satisfaction decreased [2]. Furthermore, acute mental health impacts in response to the pandemic were found in an Australian study with 5070 participants, which reported COVID-19-related fears, elevated levels of psychological distress (e.g., depression, anxiety, alcohol use, loneliness, or stress), and precautious behaviors [3]. In line with the aforementioned results, a nationally representative study from the United Kingdom (N = 14,393) showed that the prevalence of mental health problems (measured with the General Health Questionnaire, GHQ-12) had increased in all examined sociodemographic groups since the outbreak of the pandemic [4]. The results from Germany pointed in the same direction. In the general population, significantly increasing problems in relevant dimensions of mental health during the pandemic were detected in Germany, for example, regarding generalized anxiety, depression, or pandemic-related anxieties [5,6]. There is also research from the early stages of the SARS-CoV-2 pandemic in which a significant increase in depression and generalized anxiety symptomatology could not be empirically detected when longer periods of comparisons were taken into account regarding the aforementioned studies [7].

Stress or burdens can be interpreted as the entirety of influences (e.g., biological, mental, and social) that induce reactions from organisms. The level of strain (e.g., physical, psychological, and behavioral) resulting from stressors depends on an individual’s biological, psychological, and social resources [8,9]. In this regard, it is important to be aware that pandemic-related psychological burdens are unevenly distributed among different subgroups of the general population. There is evidence that the implementation of lockdown restrictions to mitigate the spread of SARS-CoV-2 was associated with increased levels of depression and anxiety, which varied in intensity depending on sociodemographic variables. Even recovery from mental health problems differed between subgroups when lockdown restrictions began to be eased. For example, 18–34-year-old participants in a study from April to June 2020, when lockdown restrictions were eased, recovered from existing mental health problems at a mean of 9.5%, whereas 50–64-year-old participants recovered only 2.9% during the same period [4]. The risk factors for mental health problems due to pandemic-related lockdowns were younger age, being female, or living with young children [10].

Being clinically/physically vulnerable to developing complications in the case of a COVID-19 infection seemed to increase mental health problems. Carriers of risk factors had higher levels of mental health problems during the pandemic compared with persons without risk factors [4]. Perceived risk theory [11] offers a framework for categorizing hazards and describes how risk perception is structured. In this framework, risks are described by two factors: dread risk and unknown risk (both containing different subdimensions). On the one hand, the SARS-CoV-2 pandemic could be described as a dreadful, uncontrollable, global, and involuntary risk. On the other hand, the SARS-CoV-2 pandemic appeared to be a new risk with delayed effects, which is not easily observable. In this respect, the pandemic can be considered problematic by both factors; therefore, the resulting risk perception can be considered to be high on average. Individual risk perception might have been even more complex, considering that each person had a more or less known set of risk factors for a severe course of COVID-19. This is relevant since being a carrier of one or more of these risk factors seemingly fueled the emergence or levels of psychological burdens, as described [4].

A noteworthy fact with regard to the sphere of school life is that in March 2020, about 1.5 billion students worldwide—as well as their teachers—were absent from schools as a result of measures to contain the spread of the SARS-CoV-2 virus [12]. Due to closures, teachers in many schools were forced to teach their lessons entirely using digital tools for the first time. Besides the required changes in the teaching mode during periods of open schools, a multitude of measures to mitigate the spread of SARS-CoV-2 were taken that affected teachers’ work lives by expanding and complicating tasks. Together with many other organizational changes in teachers’ day-to-day work since the pandemic, the amount of—often abrupt—changes demanded many adjustments, which increased the burnout risk for teachers [13]. With approximately 800,000 to 850,000 teachers in Germany, teachers are a huge occupational group; therefore, targeting teachers’ health is relevant to public health. Teachers’ health importance is leveraged when taking the number of students into account: teachers are responsible for between 9,000,000 and 10,000,000 individuals [14]. Teachers’ health is highly relevant to the socioeconomic system in Germany, too, due to the high number of people dependent on teachers being able to work. During the pandemic, school closures created a cascade of consequences for parents, employees, and companies, since parents had to take care of the children during the otherwise school time. Another important argument for researching teachers’ mental health, especially during the SARS-CoV-2 pandemic is that they work in an environment with a high number of interactions per day. At a time when interaction could mean transmission of the SARS-CoV-2 virus, this could have felt like a burden for teachers. Besides these tangible reasons for conducting research on teachers, there are superordinate ones, too. In Germany, we live in a society broadly dependent on the economic output of well-educated people since the possibility of relying on selling natural resources is limited in comparison with other countries. Following that line of thought, well-socialized and educated children are one of the, if not the most important, resources for our society. Beyond other aspects, healthy teachers play a key role in safeguarding and expanding the standard of living. The allocation of resources to research and derived interventions regarding teacher’s health therefore seems to be an enterprise that serves multiple worthwhile purposes simultaneously (e.g., better health, education, or prosperity). Especially in Germany, teachers’ health is more than just scientifically interesting—it is vital.

As stated before, the pandemic’s impacts differed between subgroups in the general population. For example, subgroups of university students showed more mental health problems during the pandemic than others (e.g., women vs. men) [15]. We were highly curious to discover the picture that a data sample of teachers in Germany would give us about the distribution of psychological burdens among subgroups of teachers. This might even be more relevant since there are opposing results indicating that there were no significant differences in mental health outcomes regarding gender or age in teachers [16]. Based on our experiences in supportive and consulting work with teachers and schools during the pandemic, we believe it is plausible to assume that the implementation of pandemic-related measurements and changes came with distinctive problems across school types. If one imagines distance learning or the implementation of hygienic measures in schools, it becomes clear that, for example, elementary schools with close to kindergarten-aged children can be a quite different scenario compared with special needs schools or high schools. Therefore, the additional strains and resulting stress for teachers could also potentially vary. We see our study closing a research gap.

Given the high relevance of teachers in Germany, the present study aimed to (i) investigate the status quo of the psychological burdens of teachers during the SARS-CoV-2 pandemic. We further aimed to (ii) identify subgroups of teachers with higher risks of psychological burdens (e.g., groups divided by gender, age, or risk factors for a severe course of COVID-19). These results for this subgroup analysis may provide a basis for the development of evidence-based and subgroup-specific interventions for the treatment of psychological burdens in German teachers.

## 2. Method

### 2.1. Procedure and Study Sample

Between 1 March and 31 March 2021, teachers from all federal states in Germany were invited to participate in an online survey. The participants were recruited with the support of governmental (Ministry of Education in Rhineland-Palatinate) and non-governmental institutions (Education and Science Workers’ Union), teacher-related societies (German Teachers Association), and projects associated with education (Monitor Lehrerbildung). There was a non-monetary incentive (EUR 2000.00 donation to the German Children’s Fund) to foster the willingness to participate. Informed written consent was obtained at the beginning of the online survey. The ethical committee of the Medical Association of Rhineland-Palatinate approved the study before it was conducted (application number: 2020-15531). 

### 2.2. Questionnaire and Measures

Participants completed an online questionnaire with approximately 350 items (presented in the online survey tool LimeSurvey), covering a wide range of topics, which were arranged under the following categories: (1) sociodemographic and workplace information; (2) identification of SARS-CoV-2-specific stresses and challenges in schools for teachers; (3) implementation, communication, and compliance with hygiene policies/plans, both general and school-based; (4) impact of school operations during the SARS-CoV-2 pandemic on teachers; and (5) collection of examples of proven interventions and derivation of recommendations for schools. Before being applied in the present study, the questionnaire was pretested and revised in three steps. First, experts from the Institute for Teachers’ Health and the Institute for Occupational, Social and Environmental Medicine of the University Medical Center Mainz answered and commented on the questionnaire. After revising the questionnaire, we asked the teachers to take part in a comprehensive probing for the exact understanding and associations of all items. We did this to make sure that the items were understood in the way we intended or were otherwise able to collect suggestions for (mostly minor) linguistic adaptations. After the probing, the questionnaire was revised again, and our team conducted a final (linguistic and grammatical) quality check to eliminate final errors (typing errors).

**Dependent variables:** Participants were asked to complete the validated German version of the Patient Health Questionnaire 4 [17], an established instrument for the combined screening for symptoms of depression [18] and generalized anxiety disorder [19]. Burnout was measured by two items from the Maslach Burnout Inventory (MBI) [20], which represent the dimensions of (a) emotional exhaustion (*“How often do you feel burned out from your work?”*) and (b) depersonalization (*“How often do you feel you have become more callous toward people since you took this job?”*). Relative to the full MBI, this two-item solution is optimized in terms of the questionnaire economy and still provides good predictive value [21]. After both burnout items, there was a question regarding the delta relative to pre-pandemic times (*“How would you describe this aspect in comparison to before the COVID-19 pandemic?”*). A 5-point Likert scale was used, ranging from *“(1) Currently much more often than before the COVID-19 pandemic”* to *“(5) Currently much less often than before the COVID-19 pandemic.”* To measure COVID-19-associated anxieties, participants rated items on a scale from 0 (no anxiety) to 100 (powerful anxiety). The items covered the anxiety of getting infected (*“How strong is your fear of being infected with the SARS-CoV-2 virus?”*), anxiety about infecting others (“*How strong is your fear of becoming a transmitter of the SARS-CoV-2 virus yourself, that is, infecting others around you with the corona virus?”*), and anxiety about infecting close people (“*How strong is your fear of friends or loved ones becoming infected with the SARS-CoV-2 virus?*”). We were given permission to use these items, which have also been used in other studies [22,23]. 

**Independent variables:** Sociodemographic variables (sex, age, or number of persons in household), work-related variables (part-time vs. full-time, member of school administration team, or school type), and health-related variables (COVID-19 risk factors) were surveyed. Age (in years, range 18–67) was converted into four quartiles for further analysis. Following official recommendations [24] and with the involvement of medical experts, eight personal risk factors for a severe course of COVID-19 disease were identified, and items were generated. All risk factor items began with the stem question: *“Which of the currently known risk factors for a severe course of the COVID-19 disease apply to you*,” on the basis of which specific risks were added, for example, *“cardiovascular disease with severely impaired cardiac pumping function of the heart or consequential damage (e.g., heart failure or coronary heart disease)*.*”* Response options were *“yes*,*” “no*,” and *“no answer.”* The number of *“yes”* responses *was* counted and summarized on a “risk factor scale” ranging from 0 to 8. Because of the low number of participants having more than two of these severe risk factors, we combined 2–8 risk factors into a 2+ category for further analysis. Our classification of school types was based on the official classification of the federal government [25].

### 2.3. Statistical Analyses

Statistical analysis was performed using SPSS Statistics Version 27 [26]. Descriptive analyses were conducted to (i) demonstrate sample characteristics and (ii) identify subgroups of teachers with increased psychological burdens. Analyses of variance (ANOVA) and Tukey’s post hoc tests were calculated to assess the differences in psychological burdens between the subgroups. Our interpretation of the ANOVAs followed the classification of effect sizes as small (0.01), medium (0.06), and large (0.14), as suggested by Cohen [27].

## 3. Results

A total of 39,359 teachers participated in the online survey. After data cleansing, a sample of N = 31,089 was used for further analysis. Of the participants, 77.5% were female, 22.0% male, and 0.4% diverse. The average age of the participants was 45.8 years (± 10.5). Detailed sample characteristics are displayed in Table 1. An overview of mean values and standard deviations for the dependent and independent variables, as well as the results for differences in subgroups of teachers (ANOVA), can be found in Table 2.

### 3.1. Level of Burdens

Teachers with two or more risk factors showed the highest mean value for **depression symptoms** (M = 2.34, SD = 1.65), whereas teachers working in special needs schools showed the lowest (M = 1.61, SD = 1.34). Regarding **generalized anxiety**, again, teachers with two or more risk factors for a severe course of COVID-19 showed the highest burdens (M = 2.59, SD = 1.89), whereas the lowest burdens were found in male teachers (M = 1.60, SD = 1.52). **Emotional exhaustion** was highest in teachers with two or more risk factors for a severe course of COVID-19 (M = 3.33, SD = 1.80) and lowest in teachers working in special needs schools (M = 2.29, SD = 1.66). The **pre/during pandemic change in emotional exhaustion** was biggest for teachers in primary schools (M = 3.91, SD = 0.86) and lowest for teachers in special needs schools (M = 3.58, SD = 0.89). **Depersonalization** was highest among teachers with two or more risk factors for a severe course of COVID-19 (M = 1.47, SD = 1.82) and lowest for teachers at special needs schools (M = 0.79, SD = 1.34). The **pre/during pandemic change in depersonalization** was biggest for teachers with two or more risk factors for a severe course of COVID-19 (M = 3.39, SD = 0.83) and lowest for teachers working in special needs schools (M = 3.21, SD = 0.60). The **anxiety of getting infected with SARS-CoV-2** was highest in teachers with two or more risk factors for a severe course of COVID-19 (M = 67.52, SD = 26.52), whereas members of the school management team showed the lowest anxiety (M = 45.62, SD = 27.84). The highest **anxiety of transmitting SARS-CoV-2 to others** was found in the first age quartile of teachers (18–30 years, M = 75.10, SD = 24.42), whereas the lowest anxiety was found in the fourth quartile of teachers (56–67 years, M = 54.59, SD = 30.31). The highest **anxiety of friends or loved ones becoming infected with SARS-CoV-2** was found in the first age-quartile of teachers (18–30 years, M = 71.06, SD = 25.32), and the lowest anxiety was found in male teachers (M = 56.67, SD = 29.43).

### 3.2. Subgroup Differences

The biggest differences in the mean values of **depression symptoms** were found when the number of risk factors for a severe course of a COVID-19 disease was used to divide into subgroups: F(2, 21348) = 72.18, *p* < 0.001, η^2^ = 0.007. Tukey’s post hoc test revealed significant differences (*p* < 0.05) between mean values of depression symptoms for subgroups of risk factors. Mean values increased from no risk factor to one risk factor (+0.28, 95%-CI[0.20, 0.35]) and no risk factor to two or more risk factors (+0.50, 95%-CI[0.37, 0.64]).

With regard to **generalized anxiety symptoms**, the biggest differences were found when divided into subgroups by gender: F(2, 21470) = 228.17, *p* < 0.001, η^2^ = 0.021. Tukey’s test revealed significant differences (*p* < 0.05) between mean values of generalized anxiety symptoms for subgroups by gender. Mean values increased from males to females (+0.58, 95%-CI[0.52, 0.64]) and from males to diverse (+0.50, 95%-CI[0.05, 0.94]).

The biggest differences in **emotional exhaustion** were found when divided into subgroups by gender: F(2, 20937) = 111.92, *p* < 0.001, η^2^ = 0.011; and risk factors for a severe course of COVID-19: F(2, 20788) = 114.19, *p* < 0.001, η^2^ = 0.011. Tukey’s post hoc test revealed significant differences (*p* < 0.05) between mean values of emotional exhaustion for males and females. Mean values increased from males to females (+0.44, 95%-CI[0.37, 0.51]). Since the effect-size for the differences between subgroups by gender was equal to subgroups by risk factors for a severe course of COVID19, Tukey’s test was conducted too. Mean values of emotional exhaustion increased from no risk factor to one risk factor (+0.45, 95%-CI[0.36, 0.54]) and from no risk factor to two or more risk factors (+0.75, 95%-CI[0.58, 0.92]).

Regarding the **pre/during pandemic change in emotional exhaustion**, the biggest differences were found between teachers at different school types: F(7, 18925) = 32.86, *p* < 0.001, η^2^ = 0.012. Tukey’s post hoc test revealed the highest number of significant differences (*p* < 0.05) between mean values of the pre/during pandemic change in emotional exhaustion for teachers working in primary schools compared with other school types. Mean values from primary school to special needs school showed the biggest difference (−0.32, 95%-CI[−0.40, −0.25]). Mean values from primary school to secondary general school showed the second biggest difference (−0.22, 95%-CI[−0.37, −0.07]), followed by primary school to secondary school (−0.18, 95%-CI[−0.25, −0.10]), primary school to comprehensive school (−0.16, 95%-CI[−0.22, −0.10]), primary school to vocational school (−0.16, 95%-CI[−0.23, −0.89]) and primary school to other school (−0.16, 95%-CI[−0.25, −0.06]). The difference in mean values from primary school to academic secondary school (−0.09, 95%-CI[−0.14, −0.03]) was the smallest.

With respect to the level of **depersonalization**, school type was the subgroup division that showed the biggest differences: F(7, 17866) = 12.44, *p* < 0.001, η^2^ = 0.005. Tukey’s post hoc test revealed the highest number of significant differences (*p* < 0.05) between mean values of depersonalization for teachers working in special needs schools compared with other school types. Mean values from special needs school to secondary school showed the biggest difference (+0.42, 95%-CI[0.25, 0.59]). Mean values from special needs school to comprehensive school showed the second biggest difference (+0.38, 95%-CI[0.23, 0.52]), followed by special needs school to vocational school (+0.36, 95%-CI[0.20, 0.52]), special needs school to secondary general school (+0.34, 95%-CI[0.06, 0.62]), special needs school to primary school (+0.30, 95%-CI[0.17, 0.43]) and special needs school to other school (+0.24, 95%-CI[0.04, 0.44]). The difference in mean values from special needs school to academic secondary school (+0.23, 95%-CI[0.09, 0.37]) was the smallest.

Differences in the **pre/during pandemic change in depersonalization** were greatest between teachers at different school types: F(7, 18038) = 7.00, *p* < 0.001, η^2^ = 0.003.

Tukey’s post hoc test revealed the highest number of significant differences (*p* < 0.05) between mean values of the pre/during pandemic change in depersonalization in teachers working in special needs schools compared with other school types. Mean values from special needs school to vocational school showed the biggest difference (+0.13, 95%-CI[0.06, 0.20]). Mean values from special needs school to primary school showed the second biggest difference (+0.12, 95%-CI[0.06, 0.17]), followed by special needs school to secondary school (+0.09, 95%-CI[0.01, 0.16]), special needs school to comprehensive school (+0.08, 95%-CI[0.02, 0.14]) and special needs school to academic secondary school (+0.07, 95%-CI[0.01, 0.13]). The differences in mean values from special needs school to secondary general school and other school were non-significant.

The number of risk factors for a severe course of COVID-19 was the subdivision of teachers in which the **anxiety of getting infected with SARS-CoV-2** was most clearly visible: F(2, 21291) = 295.14, *p* < 0.001, η^2^ = 0.027. Tukey’s post hoc test revealed significant differences (*p* < 0.05) between mean values of the anxiety of getting infected with SARS-CoV-2 for subgroups of risk factors. Mean values increased from no risk factor to one risk factor (+11.74, 95%-CI[13.16, 10.32]) and no risk factor to two or more risk factors (+17.85, 95%-CI[15.20, 20.49]).

The difference in the **anxiety of transmitting SARS-CoV-2 to others** was biggest between the four age-quartiles of teachers: F(3, 21170) = 356.25, *p* < 0.001, η^2^ = 0.048. Tukey’s post hoc test revealed significant differences (*p* < 0.05) between mean values of the anxiety of transmitting SARS-CoV-2 to others for subgroups of age quartiles. Mean values decreased from 18–30 years to 31–43 years (−5,33, 95%-CI[–7.50, –3.16]), 18–30 years to 44–55 years (−13.66, 95%-CI[−15.81, −11.52]) and 18–30 years to 56–67 years (−20.51, 95%-CI[−22.74, −18.28]).

Subgroup differences in the **anxiety of friends or loved ones becoming infected with SARS-CoV-2** were biggest between the four age-quartiles of teachers: F(3, 21161) = 143.60, *p* < 0.001, η^2^ = 0.020. Tukey’s post hoc test revealed significant differences (*p* < 0.05) between mean values of anxiety of friends or loved ones becoming infected with SARS-CoV-2 for subgroups of age quartiles. Mean values decreased from 18–30 years to 31–43 years (−3.46, 95%-CI[−5.57, −1.35]), 18–30 years to 44–55 years (−9.18, 95%-CI[−11.27, −7.09]) and 18–30 years to 56–67 years (−12.57, 95%-CI[−14.75, −10.40]).

## 4. Discussion

The present study aimed to (i) investigate the status quo of the psychological burdens of German teachers during the SARS-CoV-2 pandemic. Another goal was to analyze psychological burdens in particularly strained groups of teachers by (ii) identifying subgroups with higher risks. The results of this subgroup analysis may provide a basis for the development of evidence-based and subgroup-specific interventions for the treatment of psychological burdens in German teachers.

### 4.1. Level of Burdens and Comparisons Pre/during Pandemic and Teachers/General Population

During the SARS-CoV-2 pandemic in Germany, we detected comparatively high levels of psychological burdens of teachers. For example, comparing the depression symptoms (PHQ-2) of our teacher sample with those of a representative sample from the general population [6], female teachers (M = 1.93 vs. 1.24) and male teachers (M = 1.72 vs. 1.03) on average showed higher levels of burdens compared with females and males in the general population. The same pattern emerged when comparing the scores for generalized anxiety. In a comparison of our teacher sample with the aforementioned general population sample, female teachers (M = 2.18 vs. 1.19) and male teachers (M = 1.60 vs. 0.89) on average expressed higher levels of generalized anxiety (GAD-2) compared with the general population. Unfortunately, we had no access to during-pandemic representative data from the general population containing diverse persons for our outcome variables; therefore, comparisons are restricted to a binary level here.

Since in our study we used a two-item solution to approximate MBI burnout, comparisons with other studies were difficult. It is still possible to show the direction of the perceived change pre/during the pandemic, since teachers were asked questions directly regarding the change in emotional exhaustion and depersonalization. Regarding emotional exhaustion, the presented results show that the mean values for the delta question in all subgroups ranged above the scale mean of three, pointing in the direction of increased emotional exhaustion since the beginning of the SARS-CoV-2 pandemic. The same holds true for depersonalization. Again, all mean values ranged above the scale mean, which indicated an increase in depersonalization during the course of the pandemic.

Regarding corona-associated anxieties, the best approximation for a comparison of our data with the general population data was possible for the questions used in our study, “How strong is your fear of being infected with the SARS-CoV-2 virus?” and “Please rate your anxiety about the coronavirus” (both 0–100), which was used by Jungmann and Witthöft [22]. The respective means were 51.58 (SD 28.20) and 47.18 (SD 27.12). Teachers showed higher mean values for corona-associated anxiety than the sample from the general population [22]. The comparison is weakened insofar as different wordings were used and the surveys were one year apart (March 2020 vs. 2021). We think it is still possible to carefully interpret the higher corona-associated anxiety that the teachers expressed during the pandemic, as the higher values here line up with the higher values for the other psychological burdens mentioned. The risk of getting infected with COVID-19 most likely was lower for teachers in Germany in 2020 (when strict pandemic mitigating measures in schools were applied) relative to the general population [28]. The perceived risk, however—and therefore a potential parameter in the psychological processes of the genesis of corona-associated anxieties—seems to have been higher in teachers. This accelerated risk perception may have been amplified due to a vivid debate in the media regarding the role of schools as drivers of the pandemic.

### 4.2. Subgroup Differences

After determining the absolute level of psychological burdens and comparing them to general population samples and pre-pandemic times, the next point of interest was the analysis of subgroup differences in the dependent variables.

As the ANOVA results demonstrated, there were many significant differences in the levels of depression symptoms (PHQ-2) in all the considered subgroups of teachers. However, the differences revealed were small in terms of the effect sizes. The most accentuated difference was seen when teachers were subdivided based on the number of risk factors for a severe course of COVID-19 (η^2^ = 0.007). Most teachers did not have a risk factor for a severe course of COVID-19 (85.8%), which corresponded with fewer depression symptoms (M = 1.84, SD = 1.41) compared with having one risk factor (M = 2.11, SD = 1.55) or two or more (M = 2.34, SD = 1.65). Having risk factors was associated with more psychological burdens from depression symptoms. These included not only having to isolate oneself during the pandemic due to lockdown restrictions, but also having a severely increased risk of harm in case of an infection seemed to promote depression symptoms in teachers.

Regarding the symptoms of generalized anxiety (GAD-2), there were significant differences in all the considered subgroups of teachers. Subdivided by gender, the biggest effect on the symptoms of generalized anxiety was found (η^2^ = 0.021). This is still considered a small effect. Female teachers showed the highest mean value (M = 2.18) in the GAD-2 scores, followed by diverse (M = 2.09) and male teachers (M = 1.60). The disproportional distribution we found in our teacher sample is in line with other studies showing that females have considerably higher lifetime (1:1.9) and 12-month (1:2.2) prevalence ratios for generalized anxiety disorders [29] than men.

The levels of emotional exhaustion differed significantly in all analyzed subgroups, except for the division in quartiles of age. This result is interesting insofar as the results from a pre-pandemic study indicate that there were age-related differences in psychological burdens, such as burnout, in teachers in Germany. The highest incidence of burnout was found in teachers in the range of 50–59 years of age (6.6%) and the lowest within 18–29 years of age (1.4%) [30]. It might be that the levels of burnout in the different age quartiles were “equalized” by the SARS-CoV-2 pandemic since it fundamentally disrupted and stressed the professional and private lives of teachers equally without sparing any age group. As in depression symptoms, the most accentuated difference in emotional exhaustion was found when teachers were subdivided based on the number of risk factors for a severe course of COVID-19 (η^2^ = 0.011). Living a private life and working as a teacher during the pandemic while having a severely increased risk of harm in the case of an infection seemed to amplify the levels of emotional exhaustion in teachers. The mean values for emotional exhaustion support this perspective, since teachers with no risk factors showed the lowest (M = 2.58, SD = 1.75), teachers having one risk factor in the middle (M = 3.03, SD = 1.80) and teachers with or more than two risk factors had the highest (M = 3.33, SD = 1.80) levels of emotional exhaustion. Regarding the pre/during pandemic change in emotional exhaustion, subgroups of teachers subdivided by school type differed the most (η^2^ = 0.012) compared with the other subdivisions conducted in the study. As stated earlier, different school types were affected differently by the impact of the pandemic. One can easily imagine how different a day of teaching in an elementary school, a special needs school, and an academic secondary school might look like under normal circumstances; add extensive anti-pandemic measures and COVID-19-related uncertainties for all persons involved, and the differences between school types are unlikely to shrink.

It was noticeable that the level of depersonalization (independent of subgroups) was far below the level of emotional exhaustion, even though the items used the same wording for the answers. This may be an indication that for teachers, having good and respectful contact with their students was important and that teachers were more prone to exhaust themselves emotionally than to become cynical about their students’ needs. When assessing subgroup differences in depersonalization, only the subdivision by school management membership did not reveal significant differences. Depersonalization differed the most between school types (η^2^ = 0.005). Teachers in secondary schools expressed the highest mean values for depersonalization (M = 1.21, SD = 1.69), and teachers in special needs schools expressed the lowest (M = 0.79, SD = 1.34). Explanations for differences in depersonalization between school types are difficult to derive. One possibility arose from the accompanying qualitative research (not all school types were included) that we conducted [31]. In this context, we talked to a group of teachers from a special needs school and were therefore able to cautiously formulate the hypothesis that the lower levels of depersonalization might be because most teachers in special needs schools are working in smaller classes and are likelier to medically supervise their students and therefore may have closer relationships with students, which might keep the level of depersonalization comparatively low. Regarding the pre/during pandemic change in depersonalization, no significant differences in subdivision by number of persons in the household were found. All other ANOVAs showed significant subgroup differences, with the biggest effect for the subdivision by school type (η^2^ = 0.003). Teachers from vocational schools reported the highest (M = 3.34, SD = 0.69) and teachers from special needs schools the lowest (M = 3.21, SD = 0.60) pre/during pandemic change in depersonalization. The line of potential explanation overlaps heavily with depersonalization. To avoid redundancies, this part is not repeated.

Before discussing the subgroup results for the three corona-associated anxieties considered (getting infected, infecting others, and infection of close people) independently, it is noticeable that subdividing by working schedule (full-time vs. part-time) did not reveal significant differences in any of them. The lesson learned from that is that, independent of how many hours teachers had to work in schools during the pandemic, the levels of corona-associated anxieties were the same. Unsurprisingly, regarding the anxiety of getting infected, when subdividing by the number of risk factors for a severe course of COVID-19, teachers with 2+ risk factors (M = 67.52, SD = 26.52) exceeded the level of anxiety of teachers with one (M = 61.41, SD = 27.24) or none (M = 49.67, SD = 27.93). We think that this result is self-explanatory because it is in line with a realistic risk perception. The effect size for this subgroup comparison exceeded the size of the others (η^2^ = 0.027). The data regarding the anxiety of getting infected also showed that teachers working on the school management team were significantly less anxious compared with teachers who did not. This might be explained by the fewer hours school management team members have to spend in a classroom, where the risk perception may be more tilted toward a higher risk of infection. Another explanation could be that, by being responsible for the school and, therefore, the health of their employees and students, individual fears possibly faded from perception. The differences in the anxiety to infect others were most accentuated when subdividing by age quartiles (η^2^ = 0.048). The same holds true for the anxiety of infections of close people here, too; by subdivision in age quartiles, the most accentuated group effects were found (η^2^ = 0.020). For both anxieties, the subgroup differences considered had the same direction; that is, younger teachers reported higher levels of anxiety than their older colleagues. The decrease in anxiety about infecting others with age might be explained partly because teachers in their twenties are exposed to more risk contacts during leisure time compared with teachers who are close to retirement. The pattern that was harder to explain was a decrease in anxiety regarding the infection of people close in age. A partial explanation might lie in the order of the questions since both questions were presented in the order used in this paper. When first asked about the level of anxiety of becoming a transmitter of the SARS-CoV-2 virus, the idea of being someone who could transmit the virus might have gotten primed. Being asked about the anxiety of close people getting infected next might have unconsciously included the idea of oneself being a potential transmitter.

What surprised us was the result that teachers in special needs schools showed lower levels of psychological burdens across all variables considered. Teaching in an environment that can be described as having smaller classes and a potentially higher focus on the individual needs of students might have buffered—at least to some extent—against the burdens of the pandemic. An alternative explanation for the lower psychological burdens of teachers working in special needs schools might be self-selection. Future studies should analyze the specific factors that made teaching in special needs schools during the pandemic less psychologically burdening.

Overall, the results of the ANOVAs conducted showed that, on the one hand, significant subgroup differences were detected; on the other hand, only small effect sizes regarding subgroup differences were found. Even the biggest effect size found in our study, subdivision by age regarding the corona-associated anxiety of transmitting an infection (η^2^ = 0.048), should be considered small.

### 4.3. Limitations and Future Studies

The cross-sectional design of the present study limited its potential to describe and explain the emergence and change in the psychological burdens considered. Longitudinal monitoring of teachers’ health in Germany is a goal that we were unable to achieve. The comparisons between teachers and the general population conducted in the present study relied on relatively short (pre-/during pandemic) periods of time. Therefore, it was not possible to identify long-term trends. With the cross-sectional design comes the heightened relevance of the specific timing of the survey. Our study took place during the “third wave of SARS-CoV-2” in Germany, when pandemic-mitigating measures were in place (“lockdown light”) to counter rising infection numbers and a—at the time—relatively new and easier-to-spread virus variant of concern (B.1.1.7) [32]. This context might have exacerbated risk perception and led to the amplified psychological strains we detected. Since teachers in Germany are predominantly female, there is a gender imbalance in our sample with females (77.5%) outweighing males (22.0%) and diverse persons (0.4%). Another limiting factor regarding comparisons with other studies was the lack of data for diverse persons, since the availability of such data was—and still is—limited.

## 5. Conclusions and Recommendations

Our research can serve as a base for future studies, since it provides a broad-scale (N = 31,089) actual statement on the mental health of teachers in Germany. Furthermore, our subgroup analysis revealed details about which groups of teachers were especially burdened in different dimensions of mental health. By these attributes, our data offer considerable help to derive evidence-based interventions for teachers’ most relevant mental health challenges—tailored to specific subgroups.

To conclude, we share our thoughts regarding the question: What are the lessons learned? As our comparisons with the general population indicated, teachers in Germany showed relatively high levels of psychological burdens, which most likely were amplified during the SARS-CoV-2 pandemic. Additionally, the results of our study indicate that psychological burdens in teachers were unevenly distributed among subgroups. Therefore, it is worth diving deep into subgroup analysis and identifying especially vulnerable subgroups of teachers to enable deriving interventions that extinguish fire where it burns the brightest. To name a few, teachers with risk factors for a severe course of COVID-19 should receive special attention and support, given their elevated burdens on mental health. Since teachers working at special needs schools showed lower levels of psychological burdens than teachers at other school types, differing structural elements that promote mental health should be identified (e.g., fewer students per teacher) and transferred to other school types. More depression symptoms in younger teachers, compared with their older colleagues, indicate that countermeasures, such as offers for counselling or training in relaxation techniques, could be useful and appropriate for addressing threats to their mental health. However, in addition to specific pandemic-related psychological stresses, the classic occupational health challenges of physical, biological, and chemical burdens and their resulting strains should not be disregarded. We encourage political decision makers to take these results seriously and derive interventions accordingly. We are open to supporting such interventions with the best of our study results and knowledge. Psychologically burdened teachers are not only feeling human beings who deserve help to mitigate their suffering, they are the providers of valuable resources for a prospering future well-socialized and educated children.

## Figures and Tables

**Table 1:** Sample characteristics.

Subgroups	*n*	%	*M (SD)*
**Gender**	31,089	100%	
Female	24,099	77.5%	
Male	6851	22.0%	
Diverse	139	0.4%	
**Age (years)**	31,089	100%	45.78 (10.46)
18–30	2473	8.0%	
31–43	10,957	35.2%	
44–55	10,799	34.7%	
56–67	6860	22.1%	
**Work schedule ^a^**	30,959	100%	
Part-time	12,297	39.7%	
Full-Time	18,662	60.3%	
**School** **management**	30,981	100%	
Yes	3290	10.6%	
No	27,691	89.4%	
**Persons in** **household ^b^**	30,706	100%	2.73 (1.24)
1	4540	14.8%	
2	11,373	37.0%	
3	5538	18.0%	
4	6844	22.3%	
5+	2411	7.9%	
**School type ^c^**	27,960	100%	
Primary	9030	32.3%	
Secondary general	539	1.9%	
Secondary	2162	7.7%	
Academic secondary	5451	19.5%	
Comprehensive	4016	14.4%	
Special needs	2969	9.6%	
Vocational	2699	9.7%	
Other	1367	4.9%	
**Risk factors**	21,654	100%	0.17 (0.45)
0	18,586	85.8%	
1	2428	11.2%	
2+	640	3.0%	

^a^ Reflects the number, percentage, and mean values of participants answering the questions. The number of participants (*n*) may differ between items because responding was voluntary, and therefore not all participants answered all items. ^b^ Including the answering participant. ^c^ Multiple responses were possible; only participants with exactly one school type selected were included in the table for further analyses.

**Table 2:** Psychological burdens in different subgroups of German teachers during the SARS-CoV-2 pandemic.

Variables	PHQ-2 ^β^	GAD-2 ^β^	EE ^γ^	ΔEE ^γ^	DP ^γ^	ΔDP ^γ^	Anxiety-i ^δ^	Anxiety-t ^δ^	Anxiety-o ^δ^
	*M (SD)*	*M (SD)*	*M (SD)*	*M (SD)*	*M (SD)*	*M (SD)*	*M (SD)*	*M (SD)*	*M (SD)*
**Gender ^α^**									
F	38.84 ***	228.17 ***	111.92 ***	68.33 ***	16.54 ***	4.15 *	124.80 ***	175.31 ***	176.53 ***
η^2^	0.004	0.021	0.011	0.006	0.002	0.000	0.012	0.016	0.016
Female ^a^	1.93 (1.44) ^b^	2.18 (1.67) ^b^	2.75 (1.76) ^b^	3.83 (0.90) ^b,c^	1.04 (1.56) ^b^	3.29 (0.69) ^b^	53.19 (27.82) ^b^	65.47 (29.22) ^b^	65.52 (28.09) ^b^
Male ^b^	1.72 (1.40) ^a,c^	1.60 (1.52) ^a,c^	2.31 (1.74) ^a^	3.66 (0.87) ^a^	1.19 (1.64) ^a^	3.33 (0.70) ^a^	45.89 (28.78) ^a^	56.29 (30.53) ^a^	56.67 (29.43) ^a^
Diverse ^c^	2.25 (1.82) ^b^	2.09 (1.81) ^b^	2.75 (1.91)	3.56 (0.85) ^a^	1.12 (1.60)	3.26 (0.74)	48.70 (29.40)	58.51 (33.24)	57.82 (34.33)
**Age (years)**									
F	16.76 ***	3.06 *	1.15 (ns)	6.87 ***	21.27 ***	8.93 ***	3.82 **	356.25 ***	143.60 ***
η^2^	0.002	0.000	0.000	0.001	0.003	0.001	0.001	0.048	0.020
18–30 ^a^	2.01 (1.43) ^c,d^	2.09 (1.65)	2.63 (1.69)	3.72 (0.89) ^b,c^	1.11 (1.58) ^d^	3.30 (0.68)	52.31 (26.80)	75.10 (24.42) ^b,c,d^	71.06 (25.32) ^b,c,d^
31–43 ^b^	1.94 (1.43) ^c,d^	2.02 (1.65)	2.67 (1.75)	3.81 (0.91) ^a,d^	1.18 (1.64) ^c,d^	3.33 (0.71) ^c,d^	52.36 (28.17) ^c^	69.77 (27.81) ^a,c,d^	67.60 (27.66) ^a,c,d^
44–55 ^c^	1.88 (1.47) ^a,b,d^	2.09 (1.69)	2.67 (1.80)	3.81 (0.89) ^a,d^	1.06 (1.58) ^b,d^	3.29 (0.68) ^b^	50.88 (28.47) ^b^	61.44 (30.05) ^a,b,d^	61.88 (29.08) ^a,b,d^
56–67 ^d^	1.78 (1.40) ^a,b,c^	2.02 (1.62)	2.62 (1.79)	3.77 (0.86) ^b,c^	0.94 (1.49) ^a,b,c^	3.27 (0.67) ^b^	51.38 (28.19)	54.59 (30.31) ^a,b,c^	58.49 (29.00) ^a,b,c^
**Work schedule**									
F	12.90 ***	89.63 ***	5.15 *	2.12 (ns)	14.18 ***	14.76 ***	0.38 (ns)	0.89 (ns)	0.17 (ns)
η^2^	0.001	0.004	0.000	0.000	0.001	0.001	0.000	0.000	0.000
Part-time	1.93 (1.43)	2.18 (1.66)	2.69 (1.73)	3.78 (0.91)	1.02 (1.55)	3.28 (0.66)	51.73 (27.71)	63.18 (29.41)	63.46 (28.14)
Full-Time	1.86 (1.44)	1.96 (1.65)	2.63 (1.79)	3.80 (0.88)	1.10 (1.59)	3.32 (0.71)	51.49 (28.51)	63.58 (30.00)	63.63 (28.96)
**School** **management**									
F	61.45 ***	22.47 ***	17.12 ***	35.77 ***	1.80 (ns)	9.44 **	116.49 ***	76.89 ***	84.43 ***
η^2^	0.003	0.001	0.001	0.002	0.000	0.000	0.005	0.004	0.004
Yes	1.66 (1.40)	1.90 (1.63)	2.51 (1.79)	3.90 (0.84)	1.03 (1.50)	3.34 (0.71)	45.62 (27.84)	58.30 (31.08)	58.39 (29.84)
No	1.91 (1.44)	2.07 (1.66)	2.67 (1.77)	3.78 (0.90)	1.08 (1.59)	3.30 (0.69)	52.30 (28.16)	64.07 (29.54)	64.20 (28.43)
**Persons in** **household**									
F	17.42 ***	6.21 ***	7.43 ***	9.04 ***	3.77 **	1.52 (ns)	11.54 ***	2.98 **	2.27 *
η^2^	0.003	0.001	0.001	0.002	0.001	0.000	0.002	0.001	0.000
1 ^a^	2.07 (1.50) ^b,c,d,e^	2.17 (1.68) ^b,d,e^	2.78 (1.77) ^b,d,e^	3.78 (0.91) ^d^	1.14 (1.60) ^b,d^	3.31 (0.72)	52.01 (28.14) ^e^	62.18 (29.93) ^d^	63.87 (28.81)
2 ^b^	1.87 (1.45)^a^	2.03 (1.66) ^a^	2.64 (1.78) ^a^	3.75 (0.90) ^c,d,e^	1.04 (1.56) ^a^	3.29 (0.68)	52.39 (27.91) ^d,e^	63.52 (29.84)	63.93 (28.45) ^e^
3 ^c^	1.87 (1.45) ^a^	2.08 (1.69)	2.70 (1.78) ^e^	3.81 (0.90) ^b^	1.12 (1.60)	3.30 (0.70)	52.62 (28.37) ^d,e^	63.80 (29.78)	63.92 (28.58)
4 ^d^	1.84 (1.38) ^a^	2.00 (1.62) ^a^	2.60 (1.74) ^a^	3.85 (0.86) ^a,b^	1.03 (1.55) ^a^	3.31 (0.68)	50.35 (28.05) ^b,c,e^	64.47 (29.11) ^a^	63.33 (28.45)
5+ ^e^	1.79 (1.37) ^a^	2.00 (1.66) ^a^	2.52 (1.76) ^a,c^	3.82 (0.87) ^b^	1.10 (1.61)	3.33 (0.68)	48.05 (29.39) ^a,b,c,d^	63.02 (30.64)	61.69 (29.69) ^b^
**School type**									
F	12.26 ***	22.78 ***	26.31 ***	32.86 ***	12.44 ***	7.00 ***	7.34 **	10.97 ***	8.35 ***
η^2^	0.004	0.008	0.010	0.012	0.005	0.003	0.003	0.004	0.003
Primary ^a^	1.91 (1.45) ^f^	2.25 (1.68) ^b,c,d,e,f,g,h^	2.86 (1.79) ^b,c,d,e,f,g,h^	3.91 (0.86) ^b,c,d,e,f,g,h^	1.09 (1.60) ^f^	3.33 (0.71) ^f^	52.57 (28.23) ^f^	65.38 (29.46) ^d,f,g^	65.31 (28.48) ^d,f,g^
Secondary General ^b^	1.93 (1.43) ^f^	1.94 (1.66) ^a^	2.53 (1.77) ^a^	3.69 (0.94) ^a^	1.13 (1.63) ^f^	3.27 (0.67)	54.15 (28.79) ^f^	63.89 (29.54)	63.63 (27.44)
Secondary ^c^	1.92 (1.42) ^f^	2.00 (1.64) ^a,f^	2.62 (1.74) ^a,f^	3.73 (0.94) ^a,d,f^	1.21 (1.68) ^d,f^	3.30 (0.74) ^f^	53.82 (28.34) ^d,f,g^	62.95 (30.64) ^g^	63.49 (29.16) ^g^
Academic Secondary ^d^	1.95 (1.43) ^f,g^	2.04 (1.68) ^a,f,g^	2.61 (1.75) ^a,f^	3.82 (0.89) ^a,c,e,f^	1.02 (1.55) ^c,e,f^	3.29 (0.68) ^f^	50.97 (28.08) ^c,f^	62.24 (29.73) ^a,g^	63.34 (28.37) ^a,g^
Comprehensive ^e^	1.94 (1.44) ^f^	2.02 (1.65) ^a,f,g^	2.67 (1.76) ^a,f,g^	3.75 (0.92) ^a,d,f^	1.17 (1.62) ^d,f^	3.29 (0.71) ^f^	52.70 (28.11) ^f^	64.19 (29.67) ^g^	64.10 (28.45) ^g^
Special needs ^f^	1.61 (1.34) ^a,b,c,d,e,g,h^	1.80 (1.55) ^a,c,d,e^	2.29 (1.66) ^a,c,d,e,g,h^	3.58 (0.89) ^a,c,d,e,g,h^	0.79 (1.34) ^a,b,c,d,e,g,h^	3.21 (0.60) ^a,c,d,e,g^	48.49 (27.26) ^a,b,c,d,e^	62.50 (29.08) ^a,g^	62.09 (28.38) ^a^
Vocational ^g^	1.81 (1.47) ^d,f^	1.87 (1.64) ^a,d,e^	2.48 (1.78) ^a,e,f^	3.75 (0.85) ^a,f^	1.15 (1.60)^f^	3.34 (0.69)^f^	50.53 (28.62)^c^	59.05 (30.81) ^a,c,d,e,f,h^	60.03 (29.21) ^a,c,d,e^
Other ^h^	1.89 (1.48) ^f^	1.98 (1.64) ^a^	2.62 (1.79) ^a,f^	3.75 (0.95) ^a,f^	1.03 (1.56)^f^	3.27 (0.68)	50.87 (28.57)	62.79 (30.04)^g^	62.47 (29.54)
**Risk factors**									
F	72,18 ***	94.76 ***	114.19 ***	13.28 ***	31.53 ***	5.92 **	295.14 ***	2.76 (ns)	27.26 ***
η^2^	0.007	0.009	0.011	0.001	0.003	0.001	0.027	0.000	0.003
0 ^a^	1.84 (1.41) ^b,c^	1.99 (1.63) ^b,c^	2.58 (1.75) ^b,c^	3.78 (0.89) ^b,c^	1.04 (1.55) ^b,c^	3.30 (0.68)^c^	49.67 (27.93) ^b,c^	63.21 (29.77)	62.93 (28.80) ^b,c^
1 ^b^	2.11 (1.55) ^a,c^	2.38 (1.73) ^a,c^	3.03 (1.80) ^a,c^	3.87 (0.92) ^a^	1.21 (1.69) ^a,c^	3.32 (0.74)	61.41 (27.24) ^a,c^	64.62 (29.57)	66.76 (27.37) ^a^
2+ ^c^	2.34 (1.65) ^a,b^	2.59 (1.89) ^a,b^	3.33 (1.80) ^a,b^	3.88 (0.94) ^a^	1.47 (1.82) ^a,b^	3.39 (0.83) ^a^	67.52 (26.52) ^a,b^	64.54 (30.79)	68.23 (27.58) ^a^

^α^ (ns) = non-significant on *p* > 0.05 level, * *p*-value < 0.05, ** *p*-value < 0.01, *** *p*-value < 0.001, F = F-value, η^2^ = Eta squared, degree of freedom = number of subgroups – 1. ^β^ Depression symptoms: PHQ-2 (0–3), generalized anxiety symptoms: GAD-2 (0–3). ^γ^ Burnout items: EE = emotional exhaustion (0–6), DP = depersonalization (0–6), as well as ΔEE/ΔDP (0–5) for the change in the respective items relative to pre-pandemic times. ^δ^ COVID-19 associated anxieties items (0–100): Anxiety-i = anxiety of own infection, Anxiety-t = anxiety of transmitting infection to others, Anxiety-o = anxiety of friends of loved ones becoming infected. ^a–h^ significant differences in Tukey’s test with group(s): ^a–h^ (*p*-value < 0.05); Tukey’s test conducted only for subgroups *n* > 2.

## Data Availability

The data presented in this study are available on request from the corresponding author. The data are not publicly available due to privacy restrictions.
